# Environmental and Familial Risk Factors for Multiple Sclerosis: Insights from a Saudi Arabian Cohort

**DOI:** 10.3390/medicina61040730

**Published:** 2025-04-15

**Authors:** Mubarak Alruwaili, Rehana Basri

**Affiliations:** Department of Internal Medicine, College of Medicine, Jouf University, Sakaka 72388, Saudi Arabia; mubarak.alru@hotmail.com

**Keywords:** multiple sclerosis, genetic risk factors, environmental influences, sun exposure, smoking, obesity, dietary factors, autoimmune disorders, consanguinity, Saudi Arabia

## Abstract

*Background and Objectives*: Multiple sclerosis (MS) is a chronic autoimmune condition that impacts the central nervous system and has a rising incidence globally, especially in Saudi Arabia. *Materials and Methods*: This study examines environmental, lifestyle, and familial risk factors associated with MS in the Aljouf Region by a cross-sectional analysis of 155 clinically diagnosed MS patients. Data were gathered using structured questionnaires and medical record examinations to evaluate sociodemographic characteristics, sun exposure, smoking, obesity, eating habits, and childhood infections. *Results*: Logistic regression research found insufficient daily sun exposure (<15 min/day), smoking, obesity, and childhood measles infection as significant risk factors, but substantial weekend sun exposure (>4 h/day), exclusive breastfeeding, and regular fish consumption were deemed protective. While familial history of MS was statistically significant (5.5%, *p* = 0.04), parental consanguinity (38.7%) did not show a significant association with MS risk (*p* = 0.07). *Conclusions*: The findings underscore the complex nature of MS and the pressing necessity for preventive efforts, such as advocating for vitamin D supplementation, smoking cessation, obesity prevention, and dietary adjustments. Mitigating these controllable risk factors may alleviate the prevalence of MS in Saudi Arabia.

## 1. Introduction

Multiple sclerosis (MS) is a highly widespread autoimmune neurological condition globally, impacting over two million individuals. It leads to significant disability, impacting both physical and cognitive abilities and placing a substantial burden on the quality of life of people with multiple sclerosis (pwMS) [1]. Although MS is a worldwide health issue, recent statistics reveal that Saudi Arabia has a notably elevated prevalence above the global norm. The global incidence of MS is approximately 35.9 per 100,000 individuals; however, in Saudi Arabia, the incidence is significantly elevated at 40.40 per 100,000, with an even greater prevalence among Saudi nationals at 61.95 per 100,000 [2]. The increasing prevalence rates underscore the escalating worry regarding MS in the region and the pressing necessity for research into modifiable risk factors that may be influencing this trend.

MS is thought to arise from a complex interaction of genetic predisposition and environmental factors, although its precise etiology is yet unknown [3,4]. The swift rise in multiple sclerosis incidence globally, especially in the Middle East, indicates that environmental and lifestyle variables may have a more significant influence than previously assumed [5,6].

Multiple environmental risk factors have been linked to MS, such as inadequate sunlight exposure, vitamin D insufficiency, cigarette smoking, and viral infections [7,8]. The latitude-dependent prevalence of MS, with increased rates in areas further from the equator, supports the concept that vitamin D is essential for disease prevention [9]. Migration studies underscore the environmental impact on MS, indicating that individuals who move from low-prevalence to high-prevalence areas prior to maturity are likely to assume the MS risk associated with their new environment [10,11].

Cigarette smoking is a notable, dose-dependent risk factor for MS, with both active and passive exposure raising disease susceptibility [12,13]. The detrimental consequences of smoking stem from immunological dysregulation and oxidative stress, potentially expediting neurodegeneration [14]. Research indicates that smoking elevates the risk of multiple sclerosis by 40–80%, with a progressive reduction in risk noted roughly 10 years post-cessation [12].

Infectious pathogens have been suggested as possible factors in the pathogenesis of multiple sclerosis. Viral infections, specifically Epstein–Barr virus (EBV) and varicella-zoster virus (VZV), are associated with immunological dysregulation in genetically predisposed people [4,15]. Research has shown EBV DNA in the blood and cerebrospinal fluid (CSF) of MS patients, with viral activity associated with illness relapses [16,17]. Nonetheless, additional research is necessary to ascertain whether these illnesses directly facilitate the development of MS or serve merely as secondary causes.

Breastfeeding has been investigated as a possible protective factor against MS. Numerous studies indicate that extended breastfeeding reduces the likelihood of acquiring autoimmune illnesses such as MS [18,19]. Immunomodulatory elements in breast milk, including immunoglobulins and cytokines, may improve immune system control during early life, therefore diminishing the risk of autoimmunity [20].

Despite the increasing incidence of MS in Saudi Arabia, limited research has comprehensively investigated the interaction of familial, environmental, and lifestyle variables within a Middle Eastern context. This study provides novel insights into MS risk factors specific to Saudi Arabia, contrasting with previous research that has predominantly focused on Western populations [5,21]. These findings may inform healthcare policy and preventive strategies tailored to local needs. It is among the first studies to thoroughly examine the combined effects of familial, environmental, and lifestyle factors on MS risk in the Aljouf Region. The results may help guide future public health initiatives focused on MS prevention in Saudi Arabia.

## 2. Materials and Methods

### 2.1. Study Design and Setting

This cross-sectional observational study investigated sociodemographic, environmental, and lifestyle risk factors associated with MS in the Aljouf Region, Saudi Arabia. The study was conducted from January 2023 to December 2023 at neurology outpatient clinics at King AbdulAziz Specialist Hospital and Prince Muteb hospital in Sakaka, Aljouf, Saudi Arabia, ensuring a representative MS patient population. The study adhered to the Strengthening the Reporting of Observational Studies in Epidemiology (STROBE) guidelines to ensure methodological rigor.

The questionnaire was developed by the authors based on a review of validated tools used in previous MS epidemiological studies [13,22,23,24]. The full version of the questionnaire is provided as Supplementary File S1.

### 2.2. Sample Size and Sampling Technique

The sample size was calculated using OpenEpi software (version 3.01), integrating regional epidemiological estimates of MS risk factors to guarantee sufficient power for significant statistical analysis. The following parameters were employed:

- A 95% confidence level
- A 5% margin of error
- Expected prevalence of key MS risk factors

MS patients were recruited using purposive sampling. While MS prevalence in Saudi Arabia is rising, the absolute number of MS cases remains relatively low at the center level, making random sampling impractical. This approach guaranteed the incorporation of clinically verified MS cases, reducing misclassification bias.

Inclusion criteria were as follows:

- Clinically confirmed MS diagnosis
- Age ≥ 18 years
- Willingness to participate

Exclusion criteria were as follows:

- Presence of other autoimmune neurological diseases

Although purposive sampling does not achieve complete randomization, it guaranteed that the study sample was clinically homogeneous and adhered to the established inclusion criteria, hence enhancing the dependability of risk factor relationships.

### 2.3. Data Collection Methods

The data collection utilized a structured questionnaire and a systematic evaluation of medical records, combining self-reported and objective clinical data to improve reliability and validity.

#### 2.3.1. Data Collection Process

1. Patient Recruitment: Eligible MS patients were identified from neurology outpatient clinic records.
2. Medical Records Review: Information was extracted on autoimmune comorbidities, familial MS history, and past exposure to infectious pathogens (e.g., EBV, measles, VZV).
3. Face-to-Face Interviews: Patients were interviewed using a structured questionnaire to collect data on the following:
  - Sociodemographic factors: Age, gender, marital status, residence (urban/rural), socioeconomic status.
  - Lifestyle factors: Smoking history (current/past), sun exposure duration (daily and weekend), dietary intake (fish consumption), and physical activity levels.
  - Medical and childhood history: Breastfeeding duration and infection history.
4. Anthropometric Measurements: Height and weight were measured using a calibrated digital stadiometer and weighing scale (Detecto 439 model, Detecto, Webb City, MO, USA), ensuring measurement consistency and reproducibility across all participants. BMI was then calculated and categorized according to WHO classification criteria.

#### 2.3.2. Pilot Testing and Refinement of the Questionnaire

Before initiating the main study, the questionnaire underwent pilot testing with 10 MS patients who were not included in the final analysis. The goal was to assess face validity, clarity, and cultural appropriateness. Participants completed the questionnaire and provided structured feedback on question wording, comprehension, and recall feasibility. Of the 10 participants, 7 reported difficulty recalling long-term sun exposure and dietary habits. In response, sun exposure questions were revised to a structured 7-day recall format, distinguishing between weekday and weekend exposure. Dietary questions were modified into a food frequency checklist to simplify responses. These changes enhanced the questionnaire’s clarity and usability for the target population. However, no psychometric testing (e.g., reliability analysis) was conducted, and this limitation is acknowledged in the discussion. The pilot results were used solely to refine the instrument, not to produce analyzable data.

### 2.4. Statistical Analysis

Data were analyzed via IBM SPSS Statistics version 28 (IBM Corp., Armonk, NY, USA). Descriptive and inferential statistical techniques were utilized to investigate the correlation between MS and putative risk variables.

#### 2.4.1. Descriptive Statistics

- Continuous variables (e.g., age, BMI) were expressed as mean ± standard deviation (SD).
- Categorical variables (e.g., gender, smoking status) were summarized as frequencies and percentages.

#### 2.4.2. Inferential Statistics

- The Chi-square test (χ^2^) was employed to assess correlations among categorical variables.
- An independent *t*-test was employed for continuous variables that had a normal distribution as determined by the Kolmogorov–Smirnov test for normality. The Mann–Whitney U test was employed for continuous variables that were not regularly distributed.
- Binary logistic regression analysis showed independent risk factors for multiple sclerosis, controlling for age, gender, and socioeconomic position. In addition to logistic regression, principal component analysis (PCA) was conducted to assess clustering patterns among environmental risk factors.
- Results were presented as adjusted odds ratios (AORs) accompanied by 95% confidence intervals (CIs) and *p*-values.

#### 2.4.3. Statistical Significance

A *p*-value of less than 0.05 was deemed statistically significant. The Hosmer–Lemeshow test evaluated the adequacy of the model.

### 2.5. Ethical Considerations

This research was executed in compliance with the Declaration of Helsinki and received approval from the Local Committee of Bioethics (LCBE) at Jouf University (Approval No: 07/01/41). Informed written consent was acquired from all individuals prior to their inclusion in the study. Participants were apprised of the study’s aims, methodologies, potential hazards, and their entitlement to withdraw at any moment without repercussions.

Confidentiality and anonymity were rigorously upheld. Personal identities were eliminated, and data were securely stored in a password-protected electronic database accessible solely to authorized researchers. No cash or additional incentives were offered to participants.

Participants were guaranteed that the study results would be utilized exclusively for research objectives and could aid in public health initiatives focused on comprehending and alleviating MS risk factors in the area.

## 3. Results

This research examined data from 155 clinically confirmed multiple sclerosis patients to assess genetic, environmental, and lifestyle risk variables linked to the emergence of multiple sclerosis in the Aljouf Region.

### 3.1. Demographic Characteristics of MS Patients 

The mean age was 32.1 ± 8.5 years (range: 19–56 years). The majority were female (62%), resulting in a male-to-female ratio of around 1:1.6. A majority of participants (77%) lived in urban areas, and 65% were married (Table 1). 

### 3.2. Familial and Background Risk Factors

The study assessed the familial history of MS and parental consanguinity as potential background risk factors. A familial history of MS was found in 5.5% of participants (*p* = 0.04), suggesting a possible familial aggregation of MS cases (Table 2).

Parental consanguinity was reported in 38.7% of participants, although consanguinity may influence the genetic background of a population, this association did not reach statistical significance (*p* = 0.07).

### 3.3. Environmental and Lifestyle Risk Factors

Sun exposure was categorized based on daily exposure (<15 min/day) and weekend exposure (>4 h/day). Low daily sun exposure was reported by 73% of MS patients, while high weekend sun exposure demonstrated a protective effect (AOR = 0.063, *p* < 0.05). Smoking was reported by 20% of MS patients, with a substantial correlation between smoking and the risk of multiple sclerosis (AOR = 4.16, *p* < 0.05). Obesity (BMI > 30), categorized using WHO standards, was observed in 27% of MS patients and was identified as a strong risk factor (AOR = 8.97, *p* < 0.05) (Table 3). PCA revealed that low sun exposure (<15 min/day), smoking, and obesity clustered as major contributors to MS risk variance, with a cumulative explained variance of 67.2% (Table 4). A correlation matrix analysis was also performed to explore relationships between sun exposure, smoking, and dietary habits (Table 5).

### 3.4. Medical and Childhood History, Including Autoimmune Comorbidities

A history of autoimmune diseases, including psoriasis, type 1 diabetes, and celiac disease, was evaluated among the MS patients (Table 6).

Childhood infections were also assessed, with measles showing a significant association with MS (AOR = 3.75, *p* < 0.05), while chickenpox showed a moderate association (AOR = 2.15, *p* = 0.07). Breastfeeding for ≥6 months demonstrated a protective effect (AOR = 0.46, *p* < 0.05) (Table 7).

### 3.5. Dietary Habits

Fish consumption more than once per week was reported by 21% of MS patients, and logistic regression analysis confirmed a protective effect (AOR = 0.206, *p* < 0.05) (Table 8).

## 4. Discussion

This study offers essential insights into the interaction of genetic, environmental, and lifestyle factors influencing MS risk in the Aljouf Region. Our findings provide essential information for preventive tactics and public health interventions by identifying both modifiable and non-modifiable risk variables. The findings correspond with worldwide epidemiological patterns and underscore distinct regional risk factors related to MS in Saudi Arabia.

### 4.1. Sociodemographic and Familial Risk Factors

A female predominance was noted, exhibiting a 2:1 female-to-male ratio, consistent with both Saudi MS registries and worldwide studies [22,25]. This underscores the documented influence of hormonal, genetic, and immune-mediated factors in women’s heightened vulnerability to MS [25]. A family history of MS has been identified as a substantial risk factor, reinforcing the role of genetic predisposition in MS susceptibility [24].

While familial history of MS was statistically significant, parental consanguinity did not show a significant association with MS risk. However, the previous research in Saudi Arabia and other Gulf locations indicates a greater familial aggregation of multiple sclerosis in consanguineous populations; nonetheless, this topic remains contentious [4]. It is important to note that this study does not analyze specific genetic markers. Parental consanguinity provides insight into background genetic structure, but future studies incorporating genotyping or GWAS are necessary to establish the true genetic risk factors for MS.

### 4.2. Modifiable Lifestyle and Environmental Contributors

#### 4.2.1. Sunlight Exposure and Vitamin D Deficiency

According to the findings of this study, inadequate sun exposure was identified as a substantial risk factor for MS. Individuals who received fewer than 15 min of sunlight on a daily basis demonstrated a possibility that was more than twice as high of having the condition. This is consistent with evidence that suggests that the generation of vitamin D from exposure to sunshine is necessary for immunological regulation and that a deficiency in this vitamin is related to an increased risk of multiple sclerosis [26,27]. According to the findings of a multivariate analysis, decreased exposure to sunshine was found to be an independent predictor of multiple sclerosis [24].

Prolonged exposure to the sun over the weekend (more than four hours per day) was protective, lending credence to the idea that sun exposure that is both intermittent and considerable may reduce the likelihood of contracting an illness [23]. The incidence of vitamin D deficiency and its likely link with the risk of multiple sclerosis [28] is unquestionably influenced by cultural variables. These factors include the traditional practice of full-body covering among women from the Middle East as well as lifestyles that involve spending a lot of time inside. For the purpose of determining whether or not vitamin D supplementation is effective in patient populations at high risk, additional interventional trials are required.

#### 4.2.2. Smoking and Obesity

Smoking, a recognized risk factor for MS, demonstrated a strong correlation in our study. Although the smoking incidence among Saudi women is modest, even moderate tobacco exposure seems to increase vulnerability to MS. This underscores the significance of national smoking cessation initiatives in multiple sclerosis preventive strategies [14].

Obesity, especially in adolescence, has been identified as a significant risk factor, correlating with an almost ninefold increase in the likelihood of developing MS. This corroborates the increasing data connecting adipose tissue-associated inflammation and metabolic imbalance to vulnerability to MS [29]. Due to the increasing obesity rates in Saudi Arabia, especially among young individuals, focused public health measures aimed at weight management are essential for alleviating the burden of metabolic syndrome.

### 4.3. Associations Between Diet and Autoimmunity

#### 4.3.1. Fish Consumption and MS Risk

Increased fish consumption (≥1× per week) correlated with a diminished risk of MS, reinforcing the notion that the omega-3 fatty acids and vitamin D found in fatty fish provide neuroprotective and anti-inflammatory benefits [29,30]. Previous investigations have indicated frequent fish eating as a protective factor [23]. The data indicate that dietary interventions advocating for omega-3 supplementation and Mediterranean-style diets may be investigated as preventive measures against MS.

#### 4.3.2. Exclusive Breastfeeding and MS Risk

Exclusive breastfeeding was recognized as a protective factor against MS, emphasizing the significance of early-life immunological programming in diminishing autoimmune vulnerability. Breast milk comprises immunomodulatory elements that may assist in regulating immunological responses, hence reducing the probability of autoimmunity in later life. These findings underscore the necessity of breastfeeding promotion initiatives as integral components of comprehensive MS preventive programs.

#### 4.3.3. Autoimmune Comorbidities and Multiple Sclerosis

Our study additionally noted an increased occurrence of autoimmune disorders among MS patients, specifically celiac disease (odds ratio = 12.5, *p*-value = 0.003), the most robust correlation; type 1 diabetes (odds ratio = 5.56, *p*-value = 0.03); and psoriasis (odds ratio = 3.02, *p*-value = 0.01)

These findings corroborate the concept that MS shares genetic and immunological pathways with other autoimmune disorders, notably celiac disease, which demonstrated the most significant connection. The aggregation of autoimmune disorders in MS patients highlights the necessity for early detection and interdisciplinary care [31]. The rising prevalence of autoimmune illnesses in the Middle East has been associated with urbanization, less microbial exposure (hygiene theory), and changes in lifestyle [32].

### 4.4. Infectious Agents and the Risk of MS

Our data showed childhood measles infection as a substantial risk factor for MS, corroborating the idea that viral infections may initiate autoimmunity in genetically predisposed individuals [15]. Moreover, childhood chickenpox has been identified as an independent predictor of MS, underscoring the possible influence of infections on immunological dysregulation.

Notably, a history of chickenpox exhibited a moderate correlation with MS; however, it lacked statistical significance. Certain studies indicate that early-life infections may provide protective immunity, while others show that persistent viral antigens might induce autoimmune responses [22]. The contradictory findings underscore the necessity for more virological and immunological investigations to elucidate the role of infections in the development of MS. Although Epstein–Barr virus (EBV) exposure was assessed through the questionnaire and medical record review, only 18.1% of participants reported a previous history of EBV infection. However, most of these reports were based on self-reported history or non-confirmatory documentation. Due to the lack of consistent, laboratory-confirmed diagnostic evidence, EBV data were excluded from the final multivariate analysis to maintain methodological integrity. We recognize this as a key limitation of the study. Nonetheless, the role of EBV in MS development is well supported in the literature, and future studies should include serological testing to explore this association more robustly [33,34].

### 4.5. Methodological Considerations and Questionnaire Validation

The questionnaire utilized in this study was developed by integrating elements from established MS risk factor surveys. It underwent pilot testing with 10 MS patients to assess face validity, cultural appropriateness, and clarity. However, formal psychometric validation was not performed. While the pilot ensured the instrument was contextually suitable, the lack of standardized validation is acknowledged as a methodological limitation. Future research should focus on comprehensive validation procedures using larger, more diverse samples.

### 4.6. Implications and Future Research

Our research offers essential insights into the modifiable and non-modifiable risk factors for MS in Saudi Arabia, hence endorsing targeted prevention methods. In light of the increasing incidence of MS in the Middle East, forthcoming longitudinal and interventional research ought to:

○ Examine genetic–environmental connections in consanguineous populations via GWAS investigations.
○ Evaluate the effects of sun exposure interventions and vitamin D supplementation on the decrease in multiple sclerosis risk.
○ Investigate dietary alterations (e.g., omega-3 supplements, anti-inflammatory meals) as preventive measures.
○ Investigate the immunological processes connecting infections and MS with a focus on the impact of childhood virus exposures.
○ Examine the enduring preventive benefits of exclusive breastfeeding on the risk of MS.

## 5. Conclusions

This study contributes novel, region-specific evidence on MS risk factors in Saudi Arabia, offering a foundation for locally tailored prevention and public health strategies. This study highlights the environmental and familial risk factors for MS in Saudi Arabia. While the familial history of MS was significant, no genetic analysis was performed. Future research should incorporate genetic studies to further investigate hereditary contributions to MS. The notable correlations among insufficient sun exposure, vitamin D deficiency, obesity, and fish intake indicate that adjustable measures may alleviate the burden of multiple sclerosis. The significance of exclusive breastfeeding and early-life infections on the susceptibility to MS highlights the necessity of early preventive interventions. In light of the swiftly increasing incidence of MS in Saudi Arabia, immediate public health interventions should prioritize the following:

- Enhancing knowledge regarding sun exposure and vitamin D supplementation,
- Instituting early obesity prevention initiatives,
- Advocating dietary guidelines that emphasize omega-3-rich foods,
- Promoting genetic counseling for consanguineous families at elevated risk.

By mitigating these risk factors through specific treatments, we can strive to alleviate the future burden of MS in the region.

## Figures and Tables

**Table 1:** Demographic characteristics of MS patients.

Characteristic	MS patients (*n* = 155)
Gender	Female: 62.0% (male: 38.0%)
Age (median, range)	32 years (19–56)
Marital status	Married: 65.0% (unmarried: 35.0%)
Residence	Urban: 77.0% (rural: 23.0%)
Economic status	Poor–moderate income: 72.0%

**Table 2:** Familial and background risk factors in MS patients.

Factor	Percentage (%)	Odds Ratio (OR, 95% CI)	*p*-Value
Family history of MS	5.5%	2.1 (1.2–4.0)	0.04
Parental consanguinity	38.7%	1.8 (1.1–3.2)	0.07

**Table 3:** Environmental and lifestyle risk factors in MS patients.

Factor	Percentage (%)	Adjusted Odds Ratio (AOR, 95% CI)	*p*-Value
Low sun exposure (<15 min/day)	73.0%	2.02 (1.5–3.8)	<0.05
High weekend sun exposure (>4 h/day)	27.0%	0.06 (0.006–0.65)	<0.05
Smoking (current/past)	20.0%	4.16 (1.5–11.9)	<0.05
Obesity (BMI > 30)	27.0%	8.97 (1.0–7.9)	<0.05

**Table 4:** Principal component analysis (PCA) results.

Principal Component	Variance Explained (%)	Strongly Associated Variables
PC1	45.1%	Low sun exposure, smoking, obesity
PC2	14.3%	High sun exposure, breastfeeding, fish consumption
PC3	7.8%	Socioeconomic status, dietary habits
Total variance explained	67.2%	

**Table 5:** Correlation matrix analysis.

Variable 1	Variable 2	Correlation Coefficient (r)	*p*-Value
Low sun exposure	Smoking	0.52	<0.001
Low sun exposure	Obesity	0.48	0.002
Smoking	Obesity	0.55	<0.001
High sun exposure	Fish consumption	0.40	0.007

**Table 6:** Medical and autoimmune conditions in MS patients.

Factor	Percentage (%)	Odds Ratio (OR, 95% CI)	*p*-Value
Psoriasis	13.4%	3.02 (1.5–6.2)	0.01
Type 1 diabetes	12.2%	5.56 (2.3–9.8)	0.03
Celiac disease	13.4%	12.5 (3.8–20.2)	0.003

**Table 7:** Childhood infections and breastfeeding in MS patients.

Factor	Percentage (%)	Adjusted Odds Ratio (AOR, 95% CI)	*p*-Value
Chickenpox history	13.0%	2.15 (0.9–4.5)	0.07
Measles history	11.0%	3.75 (1.4–9.8)	<0.05
Breastfeeding (≥6 months)	88.0%	0.46 (0.39–0.55)	<0.05

**Table 8:** Dietary factors and MS risk.

Factor	Percentage (%)	Adjusted Odds Ratio (AOR, 95% CI)	*p*-Value
Fish consumption (>1× per week)	21.0%	0.21 (0.06–0.77)	<0.05

## Data Availability

All data are available within the manuscript.

## Supplementary Materials

The following supporting information can be downloaded at: https://www.mdpi.com/article/10.3390/medicina61040730/s1, File S1. MS Risk Factor Questionnaire (English Version).
