# Supplementary material for: Environmental and Familial Risk Factors for Multiple Sclerosis: Insights from a Saudi Arabian Cohort
Source: Medicina (Kaunas). 2025 Apr 15;61(4):730. doi: 10.3390/medicina61040730 (PMC12028895; doi:10.3390/medicina61040730)
Supplement: Supplementary file 1 [file medicina-61-00730-s001.zip › medicina-3512348-supplementary.pdf]

Supplementary File S1 – MS Risk Factor Questionnaire (English Version)

This questionnaire was developed to assess sociodemographic, environmental, lifestyle, and familial risk factors potentially associated with multiple sclerosis (MS) in a Saudi population.

Section S1: Sociodemographic Information

1. Age: ______ years

2. Gender:
- [ ] Male
- [ ] Female

3. Nationality: _________________

4. Place of residence: _________________

5. Education level:
- [ ] Primary
- [ ] Secondary
- [ ] University
- [ ] Postgraduate

6. Employment status:
- [ ] Employed
- [ ] Student
- [ ] Unemployed
- [ ] Homemaker

7. Monthly household income:
- [ ] < 5,000 SAR
- [ ] 5,000–10,000 SAR
- [ ] > 10,000 SAR

Section S2: Family Background

8. Do you have any first-degree relatives (parents, siblings, children) with MS?
- [ ] Yes
- [ ] No
- [ ] Don't know

9. Are your parents related by blood (consanguineous marriage)?
- [ ] Yes
- [ ] No
- [ ] Not sure

10. Do you have a family history of other autoimmune diseases (e.g., lupus, rheumatoid arthritis)?
- [ ] Yes
- [ ] No

If yes, specify: ________________

Section S3: Sun Exposure (Past 7 Days)

11. On average, how much time did you spend outdoors in sunlight per day (weekday)?
- [ ] < 15 minutes
- [ ] 15–30 minutes
- [ ] 30–60 minutes
- [ ] > 1 hour

12. On average, how much time did you spend outdoors in sunlight per day (weekend)?
- [ ] < 1 hour
- [ ] 1–2 hours
- [ ] 2–4 hours
- [ ] > 4 hours

Section S4: Lifestyle and Nutrition

15. Have you ever smoked?
- [ ] No, never
- [ ] Yes, currently
- [ ] Yes, in the past (stopped)

17. Do you consume fish regularly (past 7 days)?
- [ ] No
- [ ] 1 time
- [ ] 2–3 times
- [ ] > 3 times

19. Were you exclusively breastfed during infancy (as per your or your family's recollection)?
- [ ] Yes
- [ ] No
- [ ] Not sure

20. Please indicate your current body weight and height (as accurately as possible):

Weight: ______ kg

Height: ______ cm

(BMI will be calculated by the research team using the WHO formula: weight (kg) / height (m^2))

Section S5: History of Infections

21. Have you had any of the following infections in childhood or adolescence? (check all that apply)
  - [ ] Measles
  - [ ] Mumps
  - [ ] Varicella (chickenpox)
  - [ ] Epstein-Barr virus (mononucleosis)
  - [ ] Other: _______________
  - [ ] None of the above

22. Have you ever received a diagnosis or medical testing that confirmed EBV or other chronic infections?
  - [ ] Yes
  - [ ] No
  - [ ] Not sure
